# Supplementary material for: Pili mediated intercellular forces shape heterogeneous bacterial microcolonies prior to multicellular differentiation
Source: Sci Rep. 2018 Nov 8;8:16567. doi: 10.1038/s41598-018-34754-4 (PMC6224386; doi:10.1038/s41598-018-34754-4)
Supplement: Supplementary file 1 — Supplementary Information [file 41598_2018_34754_MOESM1_ESM.docx]

Supplementary Information for

**Pili mediated intercellular forces shape heterogeneous bacterial microcolonies prior to multicellular differentiation.**

Wolfram Pönisch, Kelly Eckenrode, Khaled Alzurqa , Hadi Nasrollahi, Christoph Weber, Vasily Zaburdaev and Nicolas Biais.

**Methods:**

**Metabolic activity in WT Ng microcolony.**

In order to assess the metabolic activity of bacteria within a microcolony we used two different dye which fluorescence correlates with different aspect of cellular metabolism^1,2^. We used fluorescein diacetate (FDA) (known to measure esterase activity) and 5-cyano-2,3-ditolyl tetrazolium chloride (CTC) (known to measure dehydrogenase activity). In both cases bacteria were allowed to form microcolonies similarly to what as been described in the main methods. Then they were treated differently:

*FDA:* Stock solution of FDA were made by resuspending 5mg of FDA in 1 ml of acetone and stored at -20°C. After microcolony formation for 3 hours 10 μl of FDA was resuspended in 500ul of PBS. After careful removal of the GCB medium, the FDA PBS mixture was added to the chamber and incubated in the dark for 10 minutes. The chamber was then washed 5 times with PBS and the microcolonies were then imaged by fluorescent microscopy using a GFP filter set.

*CTC:* Stock solution of CTC were made by dissolving the dye in pure water at 33mM and stored at -20°C. After microcolony formation for 3 hours, the GCB medium was carefully removed and 500ul of 3.3mM CTC in PBS was added to the chamber and incubated at room temperature for 30 min. The chamber was then imaged by fluorescent microscopy using a Texas Red filter set.

1. Wickström, C., Hamilton, I. R. & Svensäter, G. Differential metabolic activity by dental plaque bacteria in association with two preparations of MUC5B mucins in solution and in biofilms. *Microbiology* **155,** 53–60 (2009).

2. Stubberfield, L. C. F. & Shaw, P. J. A. A comparison of tetrazolium reduction and FDA hydrolysis with other measures of microbial activity. *J. Microbiol. Methods* **12,** 151–162 (1990).

**
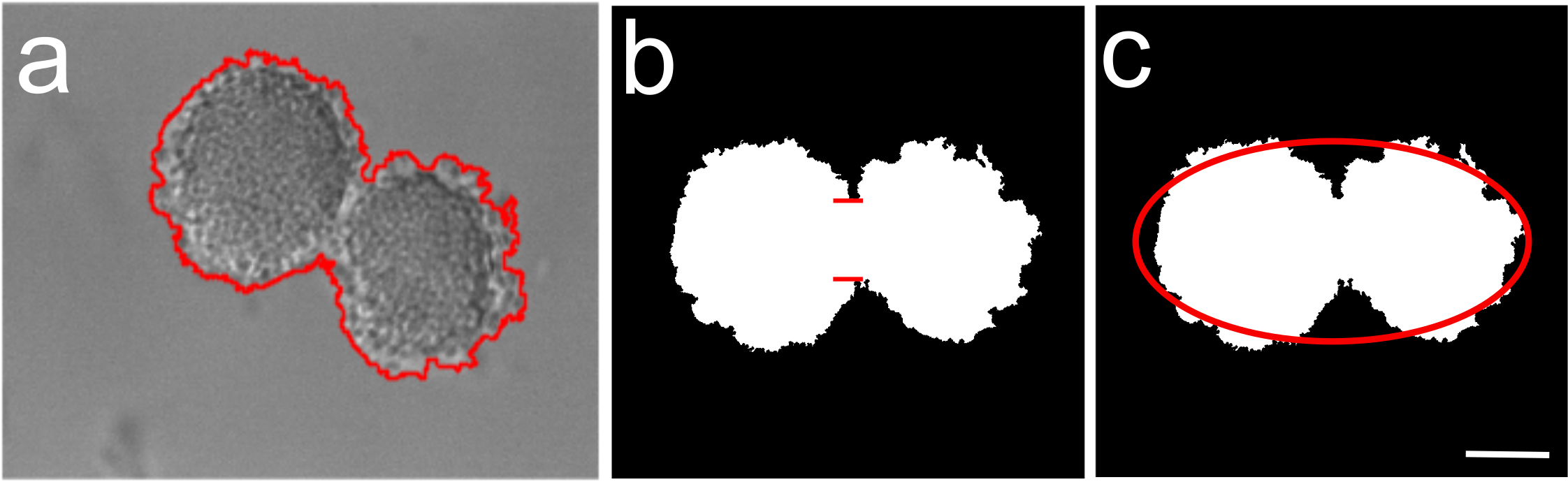
**

**Figure S1** *Analysis of Merger dynamics from experiments –* **a)** Result of the Edge Detection of the merger of microcolonies. **b)** After rotation and edge detection the bridge was measured as explained in the methods part. **c)** Ellipse fitted to the binary image of two colonies. (Scale bar = 10 µm).

**
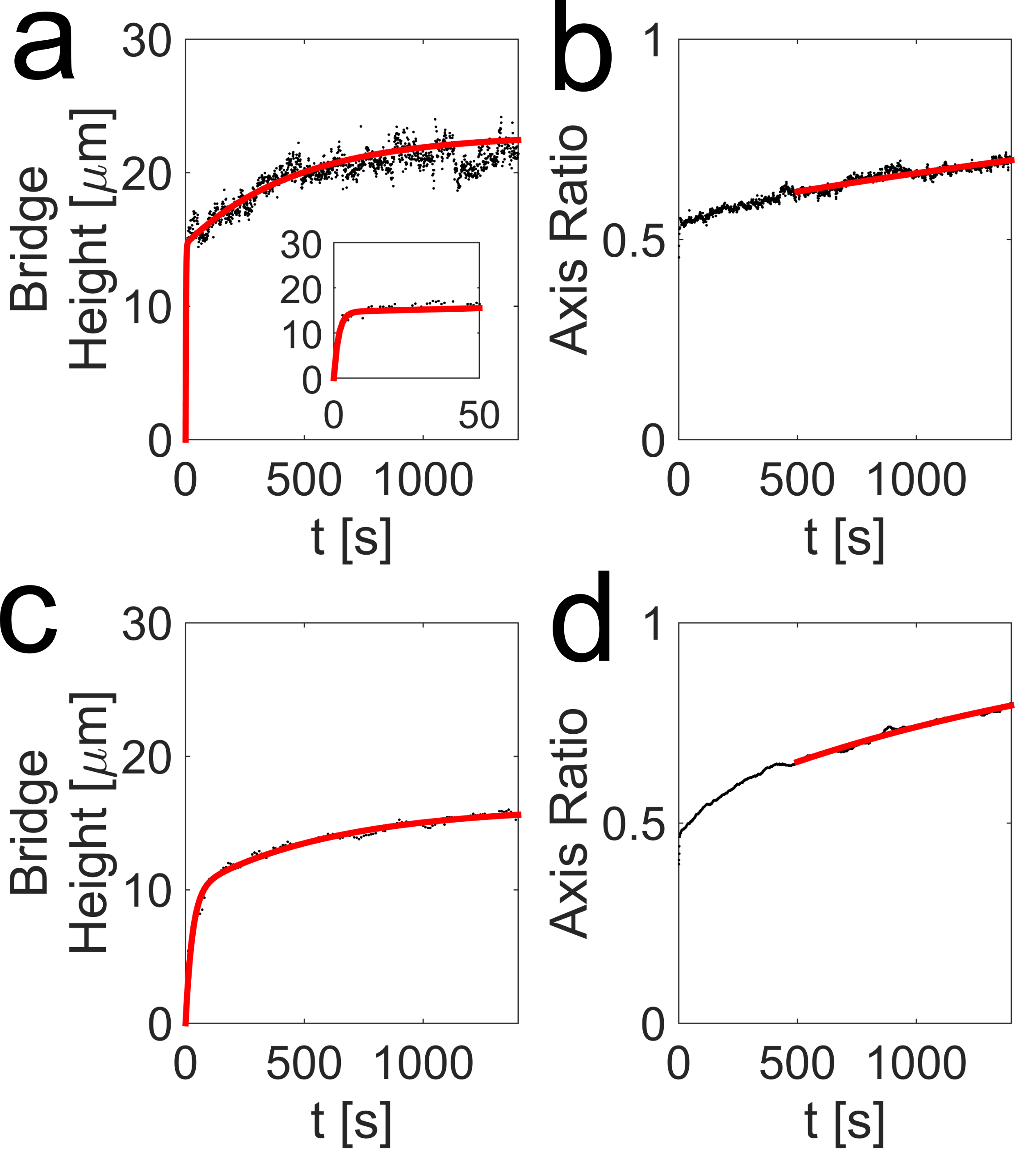
**

**Figure S2** *Time Scales of Merger –* **a)** Bridge height for experimental data. By fitting a function of the form presented in Figure2 we were able to measure the two first time scales. **b)** Axis Ratio for experimental data. By fitting we were able to compute the time scale of relaxation to a spherical shape. **c)** Bridge height for simulated merger. **d)** Axis ratio for simulated merger.

*
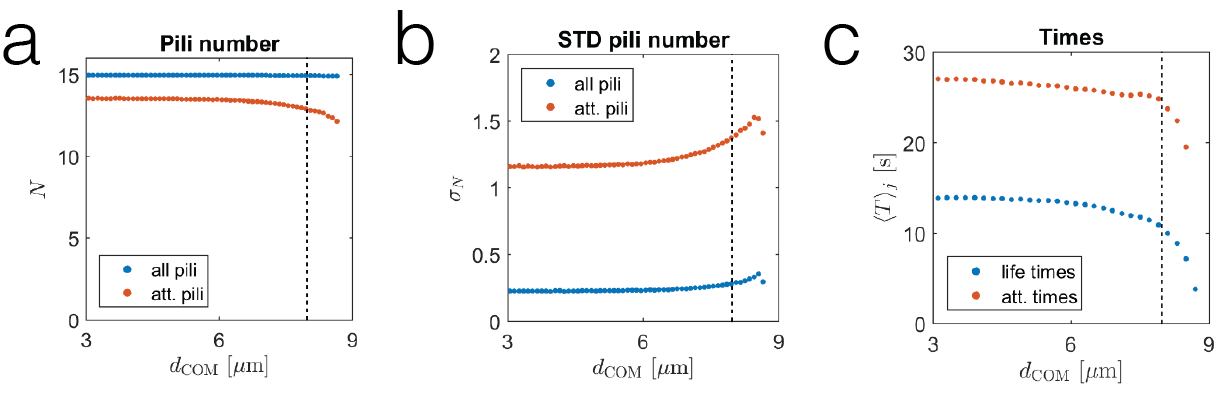
*

**Figure S3** *Number of Pili, life and attachment times for pili across a microcolony* **a)** Mean number of attached pili as a function of the distance from the center of a microcolony consisting of 850 cells. Close to the surface of the colony less pili are attached to other pili. **b)** Standard deviation of the number of attached pili as a function of the distance from the center of a microcolony consisting of 850 cells. Near the surface of the colony the fluctuations are stronger. **c)** Life times and attachment times of pili a function of the distance from the center of a microcolony consisting of 850 cells. Note that the mean life time of pili has a smaller value than the average attachment time. This discrepancy is explained by the exponential length distribution of pili, where shorter pili are less likely to bind at all and thus have a shorter life time.


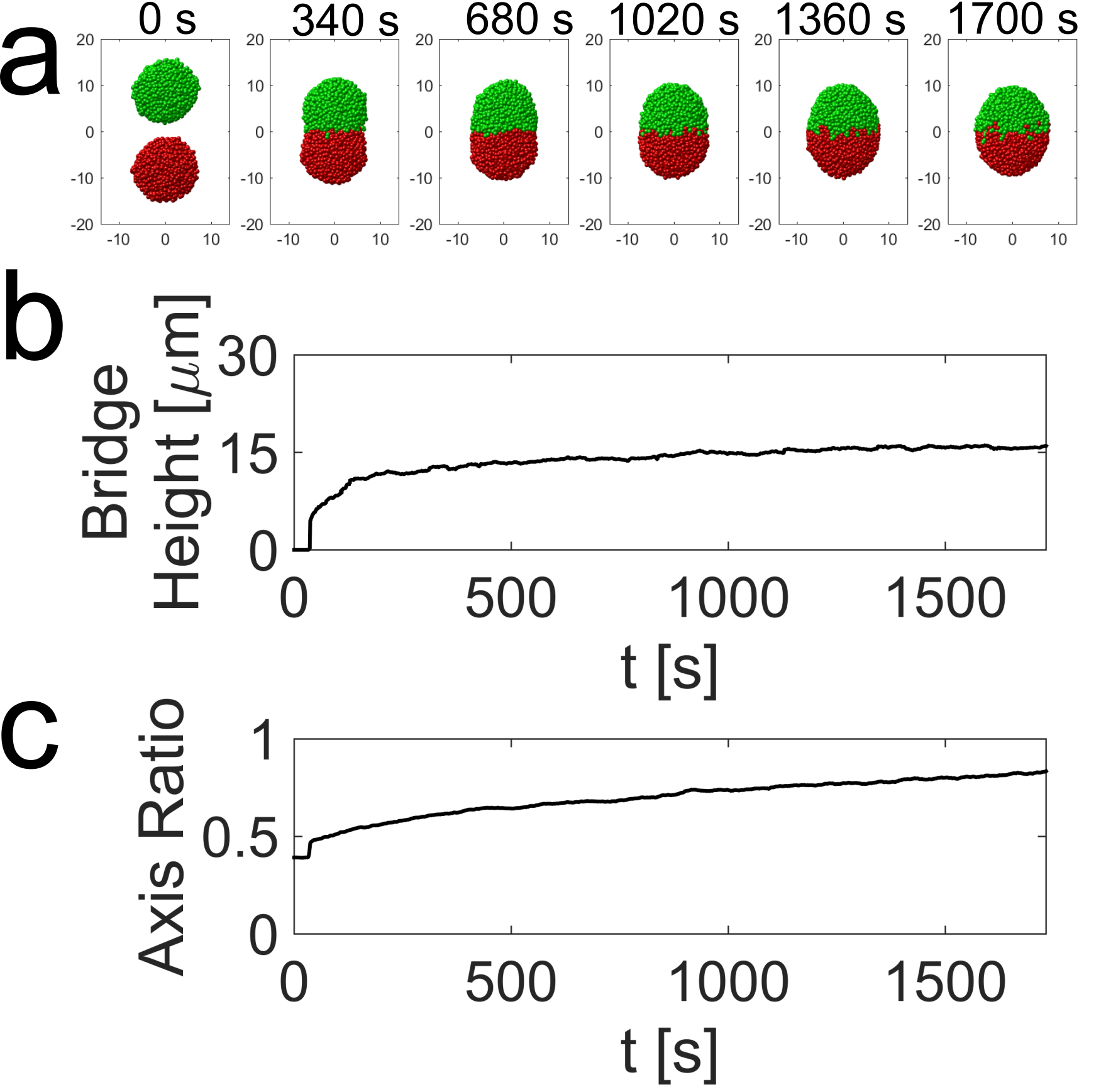


**Figure S4** *In Silico Merger –* **a)** Simulated merger. **b)** Bridge Height of the simulated data **c)** Aspect ratio of the short and long axis for the simulate data.


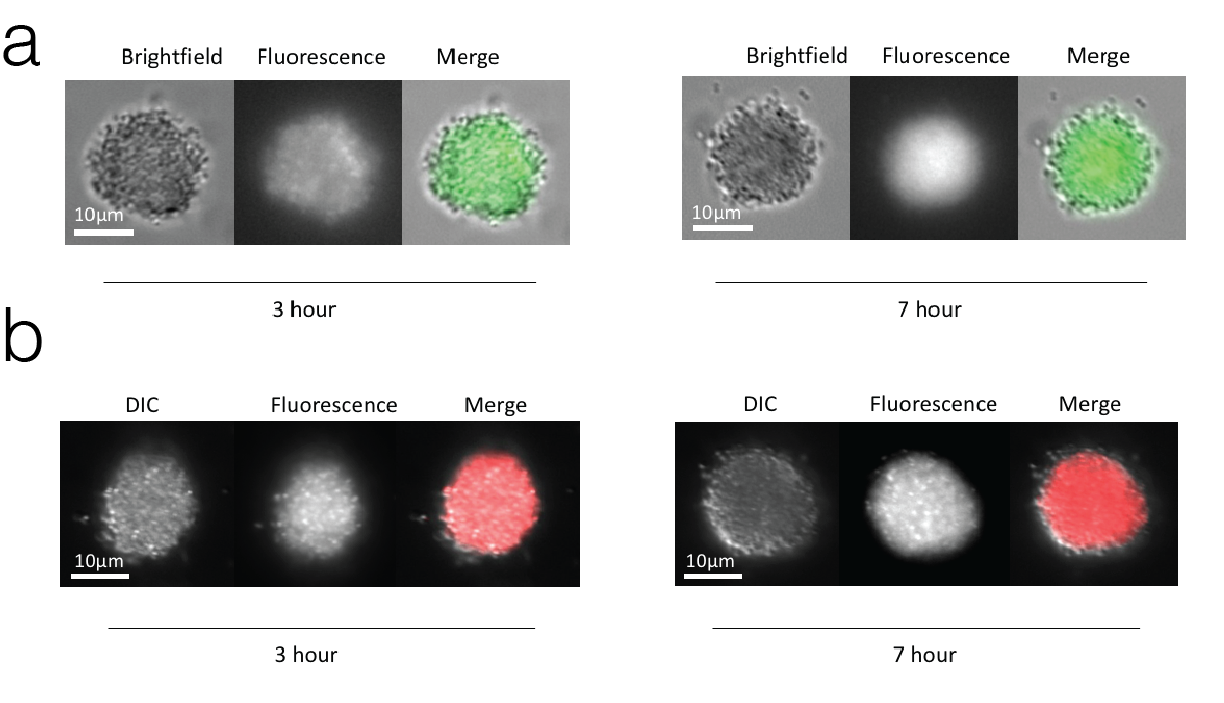


**Figure S5** *Homogeneous metabolic activity in Ng microcolonies*. **a)** Representative fluorescence image of the metabolic dye FDA in a WTNg microcolonies after 3 and 7 hours of formation. Left image is brightfield. Middle image represents the FDA fluorescence channel. The right image is the merge of both (Scale bar = 10 µm). **b)** Representative fluorescence image of the metabolic dye CTC in a WT Ng microcolonies after 3 and 7 hours of formation. Left image is brightfield. Middle image is the CTC fluorescence channel. Right Image is the merge of both (Scale bar = 10 µm)

**Supplementary MovieS1** *Formation of microcolonies by Ng bacteria*. Simultaneous DIC and fluorescence images were taken under a microscope for 3 hours as the bacteria move to form microcolonies mostly by successive merging events of smaller microcolonies (Scale bar = 20 µm).

**Supplementary MovieS2** *Merger of two Ng microcolonies.* The movie represents the merger of two Ng microcolonies where one is represented in green (YFP fluorescence) and the other in red (tdTomato fluorescence).

**Supplementary MovieS3** *Detection and* *tracking of single cells inside of microcolonies*. While DIC images were recorded with a frequency of 0.1 Hz, fluorescence images were recorded at 1 Hz. The center of mass (COM) of the microcolonies was computed from the DIC images. In order to estimate the position of the COM during the period in which no DIC images were taken, a cubic spline data interpolation applied on the the x- and y-component was used.

**Description of Theoretical Simulation Model**

# **General description**

The cell body is modeled by two spheres, called cocci, with radius $r$, positions $\vec{r}_{1}$and $\vec{r}_{2}$ and a fixed distance $d < 2r$ between the centers of the spheres to represent the diplococcus shape^1^. Individual pili protrude and retract from a fixed point on the surface of the cell.

# **Pili dynamics**

A pilus is characterized by its start and end points $\vec{r}_{\text{s}}$, $\vec{r}_{\text{e}}$ respectively. The contour length of a pilus is given by $l_{\text{c}} = \left| \vec{r}_{\text{e}}-\vec{r}_{\text{s}} \right|$**.** New pili are produced stochastically at a certain rate until a cell has a maximal number of pili $N_{\text{pili}}$. The start point of the pilus is randomly distributed on the surface of the cell (see Fig. 1). A new pilus protrudes perpendicular from the cell surface with a constant velocity $v_{\text{pro}}$. The pili switch stochastically to the retraction state and retract with the velocity $v_{\text{ret}}$. Pili do not re-elongate if retraction started. When the end of a pilus encounters the surface of the substrate, it slides over the surface. For the sake of numerical feasibility, we assume that pili can penetrate through cells. Pili are able to bind to the substrate and form binary connections to pili of other cells. In both situations, a pilus starts to retract immediately after it attached to the surface or another pilus. The binding to the substrate is also stochastic and happens at a certain rate. To model the pili-pili attachment two pili belonging to two neighboring cells are chosen randomly according to the corresponding attachments rates.


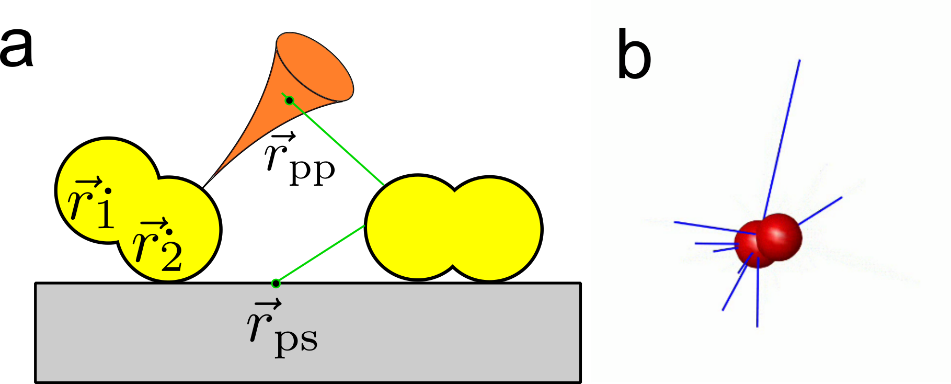


Figure 1 (a) Pili can bind to the substrate and other pili. (b) Pili of a diplococcus as computed in the simulation.

To find the coordinates of the intersection point of the two pili one pilus is assumed to sweep a conical region in space, that mimics the thermal fluctuations of an elastic rod with a certain persistence length^2^. The second pilus is modeled as a straight line. The contact point between pili is chosen randomly at the intersection segment of the line and the cone. A sketch of the attachment mechanisms is given in Fig. 1a.

Attachment of the pilus causes its contour length $l_{\text{c}}$ to deviate from its free length $l_{\text{f}}$, which is the length of the freely retracting pilus. While the pilus contour length solely depends on the motion of the cells, its free length is changing in time due to the retraction of the pilus. Pilus is modeled as a Hookean spring^3^, which gives magnitude of the pulling force of a pilus attached to the substrate ($F_{\text{ps}}$) or another pilus ($F_{\text{pp}}$) via the following relation:

$$F_{\text{ps,pp}}=\text{max}[0,k_{\text{pull}}\cdot\left( l_{\text{c}}-l_{\text{f}} \right)]$$

where $k_{\text{pull}}$ is the spring constant. Here we assume that the spring constant is high and we can neglect ist dependence on the length of the pili. The retraction velocity of the attached pilus is force dependent:

$$v_{\text{att}}\left( F \right)=\text{max}\left[ 0, v_{\text{ret}}\cdot\left( 1-\frac{F}{F_{\text{stall}}} \right) \right]$$

Here $F$ is the absolute value of the force acting on the pilus, either $\vec{F}_{\text{ps}}$ or $\vec{F}_{\text{pp}}$. $F_{\text{stall}}$ is the stalling force and is a measure for the pulling force of a pilus^4^. The pulling force also affects the detachment probability of the pilus. For the substrate-bound pili and the pili-pili-bonds the detachment rates have the standard force dependent form

$$\lambda\left( F \right)=\frac{1}{\tilde{t}}\cdot\text{exp}\left( \frac{F}{\tilde{F}} \right)$$

with $\tilde{t}=t_{\text{d,sub}}, t_{\text{d,pp}}$ and $\tilde{F}=F_{\text{d,sub}}, F_{\text{d,pp}}$ . Here $F_{\text{d,sub}}$ and $F_{\text{d,pp}}$ are the detachment forces, $t_{\text{d,sub}}$ and $t_{\text{d,pp}}$ are the detachment times for the substrate and pili interactions respectively. A detached pilus is able to rebind to the substrate or another pilus with the same rate as a growing pilus.

# **Cell Forces and Motility**

The forces in the system cause translation and rotation of the cells. An overlap of the cocci of two different cells (positions $\vec{r}_{\text{1}}$ and $\vec{r}_{\text{2}}$) causes repulsive force

$$F_{\text{cc}}=-k_{\text{cc}}\cdot\left( 2r-\left| \vec{r}_{\text{12}} \right| \right)$$

with $\vec{r}_{\text{12}}=\vec{r}_{\text{2}}-\vec{r}_{\text{1}}$ and the excluded volume spring constant $k_{\text{cc}}$. A similar equation describes the contribution of the substrate which is located at $z = 0$, resulting in the force $F_{\text{cs}}$.

The total force acting on the center of mass of the cells $\vec{r}_{\text{COM}}$ is the sum of the excluded volume forces between the cells $\vec{F}_{\text{cc}}$ and the substrate $\vec{F}_{\text{cs}}$, the forces of pili attached to the substrate $\vec{F}_{\text{ps}}$ and the forces of pili attached to other pili $\vec{F}_{\text{pp}}$, so that

$$\vec{F}_{\text{tot}}=\sum\vec{F}_{\mathrm{cc}}+\sum\vec{F}_{\text{cs}}+\sum_{\text{pili}} \vec{F}_{\text{ps}}+\sum_{\text{pili}} \vec{F}_{\text{pp}}$$

In the overdamped limit^5^, when the viscous drag forces dominate inertia (which holds for the small sizes of bacteria in water) the cell velocity is related to the force by a friction coefficient $\mu_{\text{trans}}$:

$$\frac{d}{\text{d}r}\vec{r}_{\text{COM}}=\frac{d}{\text{d}r}\vec{r}_{\text{1}}=\frac{d}{\text{d}r}\vec{r}_{2}=\mu_{\text{trans}}\cdot\vec{F}_{\text{tot}}$$

The mobility results from the Stokes friction of a spherical object in water. The rotation of a cell is described in a similar manner. The total torque is given by

$$\vec{T}_{\text{tot}}=\sum\left( \vec{r}_{\mathrm{cc}}-\vec{r}_{\text{COM}} \right)\times\vec{F}_{\mathrm{cc}}+\sum\left( \vec{r}_{\mathrm{cs}}-\vec{r}_{\text{COM}} \right)\times\vec{F}_{\text{cs}}+\sum_{\text{pili}} \left( \vec{r}_{s}-\vec{r}_{\text{COM}} \right)\times\vec{F}_{\text{ps}}+\sum_{\text{pili}} \left( \vec{r}_{s}-\vec{r}_{\text{COM}} \right)\times\vec{F}_{\text{pp}}$$

Here $\vec{r}_{\text{cc}}$ is the contact point of two cocci, $\vec{r}_{\text{cs}}$ the contact point between the cell and the substrate, $\vec{r}_{\text{s}}$ is the start point of the pilus and $\vec{r}_{\text{COM}}$ is the center of mass of the cell. The angular velocity is proportional to the magnitude of the torque

$$\omega=\mu_{\text{rot}}\cdot\left| \vec{T}_{\text{tot}} \right|$$

Here the rotational mobility $\mu_{\text{rot}}$. The angular velocity is used to rotate the cocci positions and the pili start and free pili end points around the center of mass of the cell. The above equations of motion are solved by using the simplest explicit Euler algorithm^6^ (we did check, however, that results do not change if we use higher order iteration schemes).

**Parameter Sampling**

Our model has total of 22 parameters, summarized in the Table 1. Importantly, most of parameters are known from literature and only 7 of them (highlighted in the Table 1) were used as free parameters (often with a known admissible range). The parameter set, which showed the best semi-quantitative agreement to the experimental data is provided in the Table I.

| Time step $[\text{s}]$ | $\Delta t$ | 5 · 10^−6^ |
| --- | --- | --- |
| Cocci radius $[\text{µm}]$ | $r$ | 0*.*5^1^ |
| Cocci distance $[\text{µm}]$ | $d$ | 0*.*6^1^ |
| Cell-Cell excl. vol. constant $[\text{pN/µm}]$ | $k_{\text{cc}}$ | 10^4^ |
| Cell-Sub excl. vol. constant $[\text{pN/µm}]$ | $k_{\text{cs}}$ | 2 · 10^4^ |
| Transl. friction coeff. $[\text{µm/(pN · s)}]$ | $\mu_{\text{trans}}$ | 1 |
| Rotat. friction coeff. $[\text{1/(pN · s)}]$ | $\mu_{\text{rot}}$ | 2 |
| Characteristic pili length $[\text{µm}]$ | $l_{\text{ch}}$ | 1*.*5^1^ |
| Pili persistence length $[\text{µm}]$ | $l_{\text{p}}$ | 5*.*0^7^ |
| Pili production rate $[\text{1/s}]$ | $\lambda_{\text{0}}$ | 15 |
| *Maximal pili number | $N_{\text{pili}}$ | 15 ^1,8^ |
| Pili protrusion velocity $\text{[µm/s]}$ | $v_{\text{pro}}$ | 2^1^ |
| Pili retraction velocity $\text{[}\text{µm/s]}$ | $v_{\text{ret}}$ | 2^1^ |
| Pili retraction rate $[\text{1/s}]$ | $\lambda_{\text{ret}}$ | 1.33^1^ |
| *Pili substrate attachment rate $[\text{1/s}]$ | $\lambda_{\text{sub}}$ | 0*.*5 |
| *Pili Pili attachment rate $[\text{1/s}]$ | $\lambda_{\text{pil}}$ | 0*.*5 |
| Pili spring constant $\text{[}\text{pN}\text{/µm]}$ | $k_{\text{pull}}$ | 2000^3^ |
| Stalling force $\text{[}\text{pN}\text{]}$ | $F_{\text{stall}}$ | 180^8^ |
| *Substrate detachment time $[\text{s}]$ | $t_{\text{d,sub}}$ | 10 |
| *Substrate detachment force $\text{[}\text{pN}\text{]}$ | $F_{\text{d,sub}}$ | 180 |
| *Pili-Pili detachment time $[\text{s}]$ | $t_{\text{d,pp}}$ | 50 |
| *Pili-Pili detachment force $\text{[}\text{pN}\text{]}$ | $F_{\text{d,pp}}$ | 360 |

*TABLE I. Parameter set used if not stated otherwise. The stars mark the parameters for which we performed search for optimal values. All other parameters were taken from previous studies.*

## **Single Colonies and Merger**

To find the best matching parameter set we simulated 30 minutes worth of the merging and single colonies for each parameter set, given in Table II.


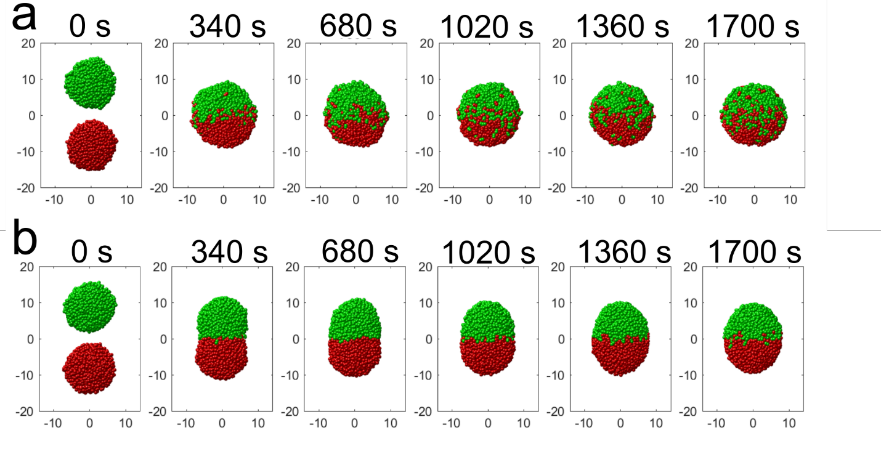


Figure 2 Example of a merger for two different parameter sets: (A) $N_{\text{pili}}=15$, $\lambda_{\text{pil}}=0.5$, $t_{\text{d,pp}}=20 \text{s}$, $F_{\text{d,pp}}=180 \text{pN}$ (B) $N_{\text{pili}}=15$, λ_pil_ = 2, t_d,pil_ = 50 s, F_d,pil_ = 360 s

| Cells – Single Colony | $N$ | 1700 |
| --- | --- | --- |
| Cells - Merger | $N$ | 1000+1000 |
| *Maximal pili number | $N_{\text{pili}}$ | 10, 15 |
| *Pili Pili attachment rate $[\text{1/s}]$ | $\lambda_{\text{pil}}$ | 0.25, 0*.*5, 2 |
| *Pili-Pili detachment time $[\text{s}]$ | $t_{\text{d,pp}}$ | 5, 20, 30, 40, 50.60, 70 |
| *Pili-Pili detachment force $\text{[}\text{pN}\text{]}$ | $F_{\text{d,pp}}$ | 120, 180, 240, 300, 360 |

*TABLE II. Parameter range for the sampling of single colony and merger simulations.*

While low detachment times, detachment forces, pili numbers and attachment rates enhanced the motility of the cells inside of the colonies and accelerated the merging, higher values reduced the motility and slowed down the merging (see Fig. 2).

## **Assembly**

By estimating four of seven free parameters from the merger and single colony simulations we performed simulations of the assembly of cells on a substrate for the remaining three free parameters (see table III). For every set of parameters one simulation was performed, as shown in Fig. 3, reproducing a total time of 1.5 hours. For a wide range of parameters we observe the demixing of ∆pilT mutants and WT cells.


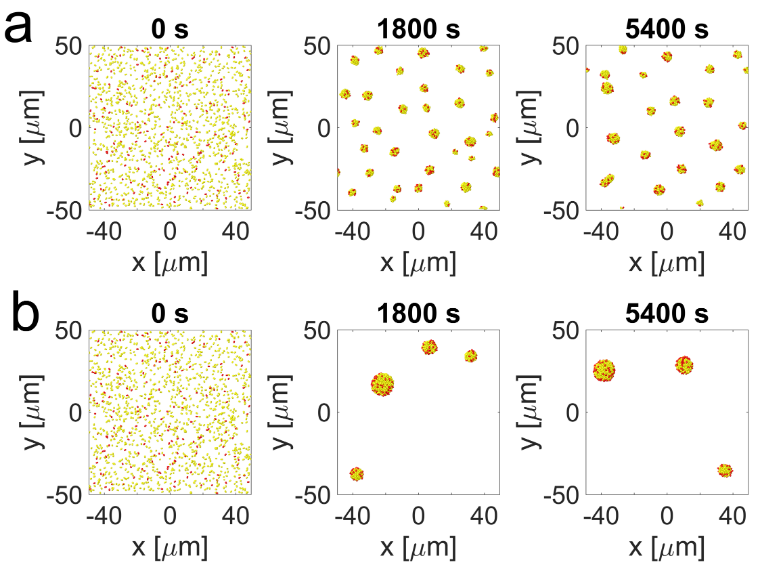


*Figure 3 Example of assembly for two different parameter sets: (A)* $N_{\text{pili}}=15$*,* $\gamma= 0.1$*,* $\lambda_{\text{sub}} = 0.5$*,* $t_{\text{d,sub}}=60 \text{s}$*,* $F_{\text{d,sub}}=300 \text{s}$ *(B)* $N_{\text{pili}}=15$*,* $\gamma= 0.1$*,* $\lambda_{\text{sub}} = 0.5$*,* $t_{\text{d,sub}}=10 \text{s}$*,* $F_{\text{d,sub}}=180 \text{s}$

| Cells Number | $N$ | 1500 |
| --- | --- | --- |
| Box Size $\left[ \text{μm×μm} \right]$ |  | 98.56$\times$98.56 |
| Percentage $\Delta$pilT | $\gamma$ | 0, 0.1, 05 |
| *Pili substrate attachment rate $[\text{1/s}]$ | $\lambda_{\text{sub}}$ | 0.25, 0*.*5, 2 |
| *Substrate detachment time $[\text{s}]$ | $t_{\text{d,sub}}$ | 0.1, 10, 30, 60 |
| *Substrate detachment force $\text{[}\text{pN}\text{]}$ | $F_{\text{d,sub}}$ | 2,180, 300, 400 |

*TABLE III. Parameter range for the sampling of single colony and merger simulations.*

Literature

1. Zaburdaev, V. *et al.* Uncovering the Mechanism of Trapping and Cell Orientation during Neisseria gonorrhoeae Twitching Motility. *Biophys. J.* **107,** 1523–1531 (2014).

2. Howard, J. *Mechanics of Motor Proteins and the Cytoskeleton*. (Sinauer Associates, Publishers, 2001).

3. Biais, N., Higashi, D. L., Brujic, J., So, M. & Sheetz, M. P. Force-dependent polymorphism in type IV pili reveals hidden epitopes. *Proc. Natl. Acad. Sci. U. S. A.* **107,** 11358–11363 (2010).

4. Maier, B. The bacterial type IV pilus system–a tunable molecular motor. *Soft Matter* **9,** 5667 (2013).

5. Van Kampen, N. G. *Stochastic Processes in Physics and Chemistry*. (Elsevier Science, 2011).

6. Schlick, T. *Molecular Modeling and Simulation: An Interdisciplinary Guide*. (Springer-Verlag New York, Inc., 2002).

7. Skerker, J. M. & Berg, H. C. Direct observation of extension and retraction of type IV pili. *Proc. Natl. Acad. Sci. U. S. A.* **98,** 6901–6904 (2001).

8. Marathe, R. *et al.* Bacterial twitching motility is coordinated by a two-dimensional tug-of-war with directional memory. *Nat. Commun.* **5,** 3759 (2014).

**Comment on the agreement between experiments and model:**

Currently our model provides a semi-quantitative agreement with the experimental data. Most likely the discrepancy is due to the simplification of the model allowing for one pilus to have at maximum one contact point with any other pili of other cells. While dictated by computer feasibility it leads to more “dynamic” microcolonies as compared to experiments. Multiple pili contact points in the real setting lead to the formation of active pili network with higher gradient in motility and less dynamic core of the cells. It is the interesting direction of further research to understand the biophysical properties of such a network.
